# ATG5-overexpressing MSCs improve ligament healing with an associated shift toward M2 macrophage polarization in aged rats

**DOI:** 10.1515/med-2026-1545

**Published:** 2026-10-12

**Authors:** Xiaoyu Chen, Jinmei Gao, Yao Wei, Guosheng Xing, Yu Yuan

**Affiliations:** Department of Ultrasound, Tianjin Hospital, Tianjin, China; Tianjin Orthopedic Institute, Tianjin, China

**Keywords:** mesenchymal stem cells, autophagy, aging, ligament healing, macrophage polarization

## Abstract

**Objectives:**

Autophagy is crucial for maintaining mesenchymal stem cell (MSC) function, yet its role in age-related ligament healing deficits remains unclear. This study examined the relationship between MSC autophagic activity and age-related ligament healing and evaluated ATG5 overexpression effects on autophagy and regeneration.

**Methods:**

Medial collateral ligament (MCL) injury models were established in young and aged rats. Healing was assessed using ultrasound, histology, immunohistochemistry, apoptosis assays, and biomechanical testing. Aged MSCs were engineered to overexpress ATG5 and transplanted into injured ligaments. Repair outcomes and macrophage polarization changes were evaluated.

**Results:**

Aged rats exhibited impaired ligament healing, and aged MSCs displayed reduced autophagic activity. ATG5 overexpression enhanced autophagy in aged MSCs, and transplantation of ATG5-overexpressing MSCs was associated with improved ligament repair in aged rats, including increased proliferation, angiogenesis, collagen organization, and biomechanical strength, alongside reduced apoptosis. ATG5-overexpressing MSC transplantation was also associated with lower M1 and higher M2 macrophage markers and a more anti-inflammatory cytokine profile. Reduced MSC autophagy was associated with age-related ligament healing deficits.

**Conclusions:**

Our findings support a potential relationship among ATG5-associated autophagy, macrophage phenotype, and improved ligament repair, while the causal dependence of therapeutic effects on autophagy and macrophage polarization remains to be established.

## Introduction

Ligament and tendon injuries are among the most common musculoskeletal disorders, accounting for nearly half of all such cases and placing a considerable socioeconomic and functional burden on patients [1], [2], [3]. Despite the availability of surgical reconstruction techniques, these injuries remain difficult to manage because of their intrinsically poor healing potential. In particular, inadequate tendon–bone integration after reconstruction often results in scar tissue formation rather than regeneration of the native enthesis, which contributes to high rates of surgical failure [1], 4], 5]. Aging further exacerbates these challenges, as age-related degenerative changes in the matrix structure, cellular activity, and mechanical properties result in poor healing quality and high rates of repair failure [4], 6], 7]. Together, these factors underscore the urgent need to develop effective strategies to increase the regenerative capacity of tendons and ligaments, particularly in the aged population.

Owing to their self-renewal, multilineage differentiation, immunomodulatory, and paracrine effects, mesenchymal stem cells (MSCs) have attracted considerable attention as promising candidates for promoting tendon and ligament healing [8], [9], [10]. However, these advantageous functions progressively decline with aging, resulting in compromised regenerative potential and ultimately restricting their therapeutic efficacy in tendon and ligament repair [11], [12], [13], [14]. Importantly, the regenerative function of MSCs is influenced by autophagy, a conserved intracellular process that maintains cellular homeostasis by degrading and recycling damaged organelles and proteins [13], 15]. Recent evidence suggests that autophagy not only supports MSC survival and stemness but also regulates their immunoregulatory capacity [13], [16], [17], [18]. Notably, aging is usually accompanied by a decrease in autophagic activity, which may further compromise MSC-mediated tissue regeneration [19], [20], [21].

Despite the increasing recognition of the importance of autophagy in MSC biology, its role in age-related impairment of ligament healing remains poorly understood. ATG5 is a key autophagy-related protein implicated in various human diseases [22]. Based on these considerations, we hypothesized that ATG5 overexpression would enhance autophagic activity in aged MSCs and improve ligament healing outcomes in aged rats. The present study was designed to answer the following research questions [1]: Is impaired ligament healing in aged rats associated with reduced autophagic activity in MSCs? [2] Does ATG5 overexpression enhance autophagic activity in aged MSCs and improve ligament repair? [3] Is transplantation of ATG5-overexpressing MSCs associated with changes in macrophage polarization within injured ligaments?

## Materials and methods

### Experimental design

In the present study, a total of 54 male Sprague–Dawley (SD) rats, including 18 young rats (3 months) and 36 aged rats (18 months) [23], [24], [25] were ultimately selected. All rats were purchased from Wei Tong Li Hua Experimental Animal Center (China) and maintained under standard housing conditions with free access to food and water. The experiment was divided into two phases. In each phase, an MCL injury model was established under isoflurane anaesthesia and verified by ultrasound imaging on postoperative Day 14. A 1-cm medial incision was made along the stifle joint to expose the MCL, which was then transected at its midpoint. The ligament was microsurgically repaired using 6–0 Prolene sutures. After wound closure, the operated limbs were supported with a soft bandage, and the rats were allowed unrestricted cage activity. Prophylactic antibiotics were administered postoperatively. On postoperative Day 14, the rats were euthanized, and the MCL specimens were harvested for further analyses. All animal procedures were conducted in compliance with institutional animal care guidelines and approved by the medical ethics committee of our hospital.

In Phase I, a total of 18 young rats and 18 aged rats were used. 12 young rats and 12 aged rats with comparable body weight were randomly selected for surgical ligament transection and repair. The right MCL of every rat undergoing surgery was transected and repaired as described above, while the contralateral MCL was left intact to serve as an internal control. The remaining 6 young rats and 6 aged rats were used for bone marrow–derived MSC isolation, and generation of autophagy-enhanced MSCs (MSC^ATG5+^ and MSC^vector^).

In phase II, an MCL injury model was subsequently established using only 18 aged rats. Following ligament repair, a small periligamentous pocket was created at the injury site. Stably transduced bone marrow–derived MSCs at passages 6–10 (1 × 10^6^ cells suspended in 20 μL PBS) were locally injected into the pocket [26]. To minimize individual variability and allow comparison under identical systemic and postoperative conditions, the right MCL received MSC^ATG5+^ cells, while the left MCL received MSC^vector^ cells in bilateral treatment in the same rat. The pocket was secured with fine sutures to minimize leakage. Of these rats, 6 rats were used for histological analysis, 6 rats for biomechanical testing, and 6 for quantitative RT-PCR analysis.The surgical operator was not blinded to treatment allocation because the designated cell preparation for each limb had to be known during transplantation. Nevertheless, identical surgical procedures, injection volumes, and postoperative management were applied to both limbs. Ultrasound examinations and assessments, histological imaging and quantitative analyses, and biomechanical testing and data analysis were performed by investigators blinded to the experimental grouping.

### Haematoxylin and eosin (H&E) staining

MCL specimens were harvested and immediately fixed in 4 % paraformaldehyde for 24 h. Following fixation, the tissues were dehydrated through graded ethanol, cleared in xylene, and embedded in paraffin. Longitudinal sections (5 μm) were cut using a rotary microtome. After deparaffinization and rehydration, the sections were stained with haematoxylin for nuclear visualization and counterstained with eosin to highlight the cytoplasm and extracellular matrix. The stained slides were rinsed, mounted, and imaged under a bright-field microscope (Olympus, Japan) to assess cellularity, inflammatory infiltration, and general tissue morphology.

### Masson’s trichrome staining

For collagen assessment, paraffin-embedded ligament sections (5 μm) were deparaffinized and rehydrated using standard protocols. Masson’s trichrome staining was performed with a commercial staining kit (Servicebio, China) according to the manufacturer’s protocol. Stained sections were dehydrated, cleared, and mounted with neutral resin. Images were captured using a light microscope (Olympus, Japan), and representative fields were selected to evaluate the extent of collagen organization, matrix remodelling and repair progression.

### Immunohistochemistry (IHC)

Formalin-fixed, paraffin-embedded ligament specimens were cut at a thickness of 5 μm, deparaffinized, and rehydrated. Antigen retrieval was performed by heating the slides in citrate buffer (pH 6.0) at 95 °C for 20 min. After the slides were cooled to room temperature, endogenous peroxidase activity was quenched with 3 % H_2_O_2_, and nonspecific binding was blocked with 5 % bovine serum albumin (BSA) for 30 min. The sections were incubated overnight at 4 °C with the following primary antibodies: Ki67 (Cat#: ab279653; 1:500; Abcam), CD31 (Cat#: ab182981; 1:1000; Abcam), α-SMA (Cat#: 14395-1-AP; 1:1000; Wuhan Sanying), COLI (Cat#: YT1018; 1:100; ImmunoWay), and COLIII (Cat#: YM3123; 1:100; ImmunoWay). After being washed with PBS, the slides were incubated with HRP-conjugated secondary antibodies (Zhongshan Golden Bridge, China) for 1 h at room temperature. Signal detection was achieved using 3,3′-diaminobenzidine (DAB), followed by counterstaining with haematoxylin. Finally, the slides were dehydrated, mounted, and imaged under a light microscope (Olympus, Japan). At least five random high-power fields from each section were evaluated per animal, with mean values used for statistical analysis. Immunoreactivity was assessed using a semiquantitative histological score [27].

### TUNEL assay

TUNEL staining was performed on paraffin-embedded sections harvested from model rat MCLs on postoperative Day 14. Apoptotic cells were detected with a commercially available apoptosis detection kit (Beyotime, China) according to the manufacturer’s instructions. Cell nuclei were counterstained with DAPI, and fluorescence images were acquired under a fluorescence microscope. For quantitative analysis, at least five non-overlapping microscopic fields were randomly chosen from each section, and the percentage of TUNEL-positive nuclei was calculated.

### Biomechanical evaluation

On postoperative Day 14, the MCLs were carefully harvested from both hind limbs. Under a stereomicroscope, adjacent connective tissues were removed while preserving the native ligament attachments at the femoral and tibial insertion sites. Throughout preparation, samples were wrapped in gauze moistened with phosphate-buffered saline (PBS) and tested on the same day to prevent dehydration. For mechanical testing, the femoral and tibial bone blocks were embedded in custom fixtures using polymethyl methacrylate to ensure secure fixation. The ligament was aligned along the loading axis, and the bone ends were clamped into the grips of a universal testing machine (EZ-SX, 500 N capacity; Shimadzu, Japan). The initial gauge length used for strain calculation was 6.3 mm. A preload of 0.1 N was applied to eliminate slack, and no cyclic preconditioning was performed. Specimens were subsequently loaded to failure at a constant crosshead displacement rate of 0.1 mm/s, corresponding to a nominal strain rate of approximately 0.0159 s^−1^. Force–displacement data were continuously recorded using Trapezium X2 software (version 2.05; Shimadzu). Failure modes were visually assessed during tensile testing. A partial rupture pattern was consistently observed across samples, with portions of the ligament failing while residual fibers remained intact.Biomechanical testing and data analysis were performed by investigators blinded to the experimental grouping.

The following mechanical parameters were determined: Failure load (N): the maximum force sustained before ligament rupture; Stiffness (N/mm): the slope of the linear region of the load–displacement curve; Maximum stress (N/mm^2^): calculated as failure load divided by the ligament’s cross-sectional area (CSA); Young’s modulus (MPa): derived from the linear portion of the stress–strain curve. Prior to mechanical testing, CSA at the midsubstance was measured using digital calipers (SD500-150PRO, Sincon) at three orthogonal orientations (0°, 60°, 120°) and averaged using the elliptical approximation formula: CSA=π × (a/2) × (b/2), where a and b are the major and minor axes. The CSA value was then used to calculate maximum stress by dividing the failure load by the CSA.

### Flow cytometric analysis of bone marrow-derived MSCs

Third-passage rat bone marrow-derived MSCs were harvested and washed twice with pre-cooled phosphate-buffered saline (PBS). The cells were resuspended in PBS containing 2 % foetal bovine serum (FBS; Gibco, USA) at a concentration of 1 × 10^6^ cells/mL. The samples were divided into five groups: unstained control, CD44, CD29, CD90, and CD45, with 1 × 10^5^ cells (100 μL) in each tube. Cells were incubated for 15–30 min at room temperature in the dark with the following FITC-conjugated monoclonal antibodies (all from Thermo Fisher Scientific, USA): anti-CD44 (IM7, Cat# 11-0441-85), anti-CD29 (integrin β1, TS2/16, Cat# 11-0299-42), anti-CD90 (OX-7, Cat# MA1-80648), and anti-CD45 (30-F11, Cat# 11-0451-82). Each antibody was tested in a separate tube using a single-color staining strategy. Following incubation, cells were washed twice with PBS and resuspended in 500 μL PBS for analysis. Unstained cells and isotype-matched FITC-conjugated antibodies served as negative controls. Quantitative analysis: At least 10,000 events were analyzed per sample using a BD FACSCalibur flow cytometer. Positive marker expression is reported as percentage of total gated cells: CD44+, CD29+, CD90+, and CD45−. MSC purity was confirmed when ≥95 % of cells expressed CD44, CD29, and CD90, while <2 % expressed CD45, consistent with ISCT criteria.

### Western blotting analysis

Total protein from rat MSCs was extracted with RIPA lysis buffer (Beyotime, China) supplemented with protease and phosphatase inhibitor cocktails (Thermo Scientific, USA). Protein concentrations were determined using a BCA assay kit (Thermo Scientific, USA). Equivalent amounts of protein (20–30 µg) were separated on SDS–polyacrylamide gels and subsequently transferred to PVDF membranes (Millipore, USA). The membranes were blocked with 5 % nonfat dry milk in TBST for 1 h at room temperature, followed by overnight incubation at 4 °C with primary antibodies against LC3 (1:1000, CST, Cat# 12741), ATG5 (1:1000, Abcam, Cat# ab108327), p62 (1:1000, CST, Cat# 23214), LAMP1 (1:1000, Abcam, Cat# ab13523), and GAPDH (1:10000, Abcam, Cat# ab181602). After being washed, the membranes were incubated with horseradish peroxidase (HRP)-conjugated secondary antibodies for 1 h at room temperature. The protein bands were visualized with an enhanced chemiluminescence detection kit (Thermo Scientific, USA), and densitometric analysis was performed using ImageJ software (NIH, USA). To confirm that observed changes reflect genuine autophagic flux rather than altered protein synthesis/degradation, cells were treated with or without chloroquine (CQ, a lysosomal inhibitor commonly used to assess autophagic flux), followed by western blot analysis of LC3 and p62 expression.

### Immunofluorescence staining

For *in vitro* analysis, MSCs were seeded on sterile glass coverslips and fixed with 4 % paraformaldehyde for 20 min, followed by permeabilization using 0.1 % Triton X-100 for 10 min at room temperature. After blocking with 5 % bovine serum albumin (BSA) for 1 h, the cells were incubated overnight at 4 °C with the following primary antibodies: LC3 (1:100, CST, Cat# 12741), ATG5 (1:200, Abcam, Cat# ab108327), p62 (1:400, CST, Cat# 23214), and LAMP1 (1:50, Abcam, Cat# ab13523). On the next day, fluorescent secondary antibodies (FITC/Cy3, 1:500) were applied for 1 h at room temperature in the dark. Nuclei were counterstained with DAPI (Beyotime, China) for 5 min. Coverslips were mounted, and images were obtained using a Leica fluorescence microscope.

For tissue-based immunofluorescence, paraffin-embedded sections of the MCLs were deparaffinized, rehydrated, and subjected to antigen retrieval. After blocking with 5 % BSA, the sections were incubated overnight at 4 °C with antibodies against CD68 (1:200, CST, Cat# 97778), CD86 (1:100, Thermo Scientific, Cat# RAB02398), and CD206 (1:400, CST, Cat# 24595). The following day, fluorophore-conjugated secondary antibodies (Invitrogen, 1:500) were applied for 1 h in the dark at room temperature. DAPI was used for nuclear staining, and fluorescence images were captured under a Leica microscope. Quantitative macrophage analysis: CD68+CD86+ (M1-like) and CD68+CD206+ (M2-like) cells were counted in five random high-power fields (400 × magnification) per section. At least five non-overlapping microscopic fields from each section were randomly chosen, with means used for statistical analysis.

### Generation of autophagy-enhanced MSCs

Primary rat MSCs were plated in 6-well plates at a density of 1.2 × 10^5^ cells/well and cultured for 24 h. Once the cells reached ∼70 % confluence, they were transduced with lentiviral particles carrying the rat ATG5 coding sequence or an empty control construct (Hanbio Biotechnology Co., Ltd., Shanghai, China) at different multiplicities of infection (MOI=10, 20, 30, 40, or 50) in the presence of 5.25 μL of polybrene (2 mg/mL). The backbone vector (Ubi-MCS-3FLAG-CBh-GFP-Puro) contained GFP for visualization and a puromycin resistance cassette to enable selection. Viral input volumes were calculated on the basis of a titre of 1 × 10^8^ TU/mL. Cells were subjected to puromycin selection at 3 μg/mL for 3 days, followed by continuous culture in 2 μg/mL puromycin to maintain stable expression. GFP expression was examined 48–72 h post infection using a fluorescence microscope, and an MOI of 50 was used to determine the optimal transduction efficiency. The resulting MSCs stably overexpressing ATG5 (MSC^ATG5+^) and the corresponding control cells (MSC^vector^) were expanded in 10-cm dishes and used for subsequent ligament experiments.

### Quantitative real-time PCR

Ligament samples were collected from the site of the MCL injury on postoperative Day 14. Total RNA was purified using TRIzol reagent (Invitrogen, USA) following the manufacturer’s protocol. Complementary DNA (cDNA) was generated from equal amounts of RNA using the PrimeScript™ RT Master Mix kit (Takara, Japan). Quantitative RT-PCR was then performed with TB Green Premix Ex Taq II (Takara, Japan) on a LightCycler 480 instrument (Roche, Switzerland). The mRNA expression of immune regulation–related markers, including CD80, CD163, IL-12, IL-10 was determined. GAPDH served as the internal reference gene, and relative expression levels were calculated according to the 2^−ΔΔCt^ method. The primer sequences are provided in Supplementary Table S1.

### CCK-8 assay

MSCs were digested, counted, and seeded into 96-well plates at a density of 1 × 104 cells per well. After overnight attachment, cells were treated with different concentrations of chloroquine (CQ; MedChemExpress, HY-17589A) at final concentrations of 0, 1, 2, 5, 10, 25, and 50 μM for 4 h. At the end of treatment, 10 μL of CCK-8 solution was added to each well and incubated for 1 h at 37 °C. Absorbance was measured at 450 nm using a microplate reader.

### ELISA assay

Ligament tissues were washed with pre-cooled PBS and homogenized in PBS at a ratio of 100 mg tissue to 500 μL PBS using a cryogenic tissue grinder. The homogenates were centrifuged at 12,000 × *g* for 15 min at 4 °C, and the supernatants were collected for subsequent ELISA analysis. The levels of TNF-α, IL-6, IL-4, and TGF-β1 were measured using commercial ELISA kits (Jiangsu Kete Biotechnology Co., Ltd., China; TNF-α: MM-0180R1, IL-6: MM-0190R1, IL-4: MM-0191R1, and TGF-β1: MM-0047R1) according to the manufacturer’s instructions. Absorbance was measured at 450 nm using a microplate reader.

### Statistical analysis

All the statistical analyses were performed with GraphPad Prism software (version 9), SPSS software (version 25), and R software (version 4.1.2). The data were expressed as the mean ± standard deviation (SD). Homogeneity of variances was evaluated using Levene’s test. For comparisons between two independent groups, an independent-samples t test was applied. For comparisons between paired groups (e.g., left vs. right limbs in the same animal), a paired samples t test was used. For comparisons among more than two groups, one-way analysis of variance (ANOVA) was performed when variance homogeneity was satisfied. Post hoc multiple comparisons were conducted using the Bonferroni correction. To further control for multiple testing across the numerous study endpoints, the Benjamini–Hochberg procedure was applied separately within each biologically related outcome family using the p.adjust function in R. An FDR-adjusted p value <0.05 was considered statistically significant. The sample size (n=6 per group) was determined by referencing previous studies using similar animal models to acquire preliminary data with the minimum number of animals in compliance with the 3Rs reduction principle for animal research ethics [28], 29].

### Ethics approval

All procedures performed in the study were approved by the Animal Ethical and Welfare Committee of Tianjin Hospital (Approval No. 2023-092). Informed consent was not applicable as this study involves experimental animals.

## Results

### Successful establishment of MCL injury rat model and worse recovery of ligament function in aged rats than young rats

An MCL injury model was successfully established and verified by ultrasound imaging on postoperative Day 14 (Figure 1A). Compared with the normal MCLs (YN and AN), the injured MCLs (YI and AI) appeared thickened, with heterogeneous hypoechoic changes and occasional punctate calcifications, especially in aged rats (AI). H&E staining revealed more pronounced disarray of fibres, obvious inflammatory cell accumulation, and delayed tissue repair in AI group on postoperative Day 14, whereas mild disturbances in collagen orientation and limited inflammatory cell infiltration in YI group (Figure 1B). Similarly, Masson’s trichrome staining indicated relatively poorer fiber arrangement and a greater proportion of immature collagen fibers in AI group than YI group (Figure 1C).

**Figure 1:**
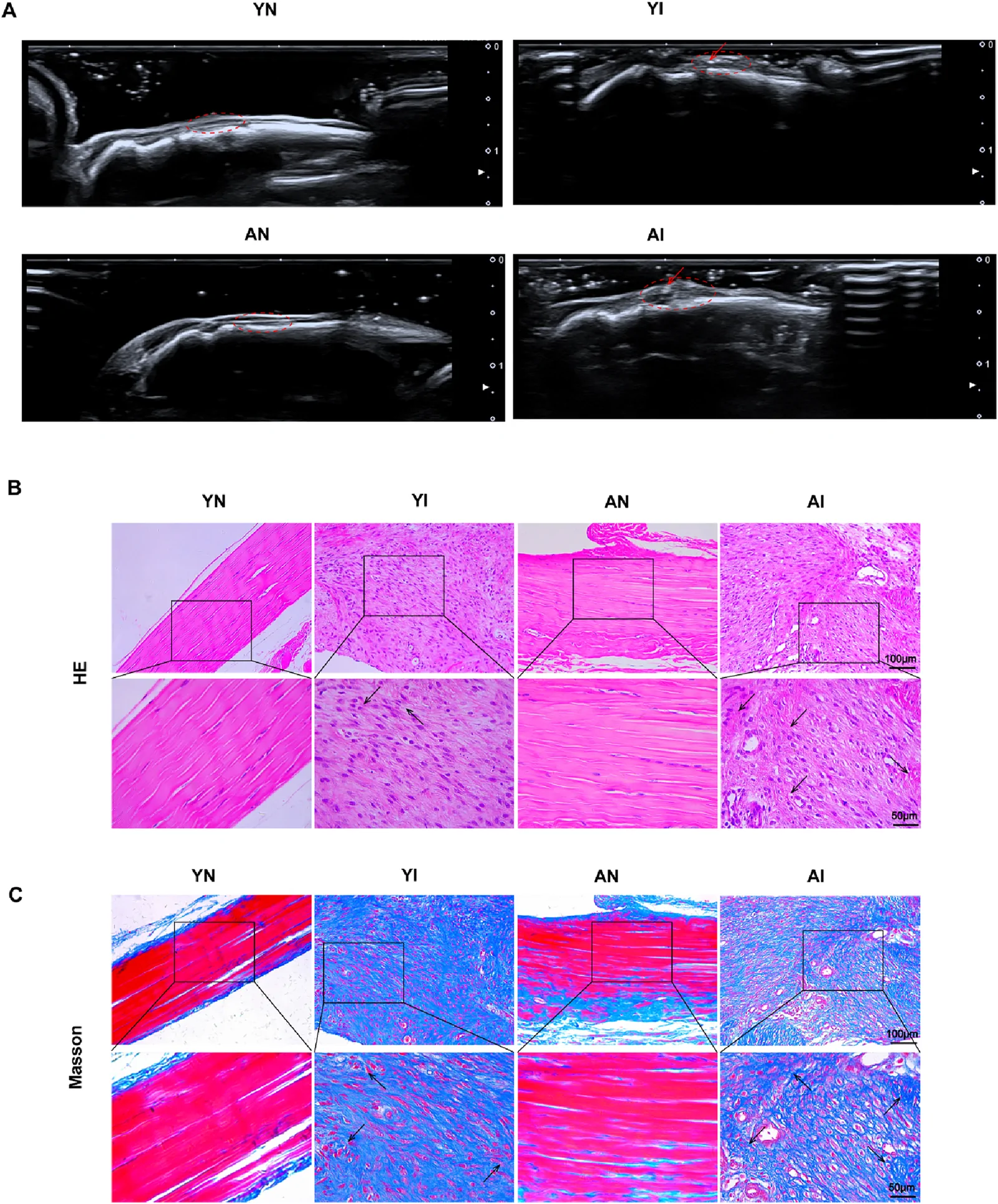
Establishment of MCL injury rat model and the function recovery of medial collateral ligament (MCL) on postoperative Day 14. (A) Representative ultrasound images; (B) haematoxylin and eosin (H&E) staining (scale bars: 50 μm; 100 μm); (C) Masson’s trichrome staining of the MCL (Scale bars: 50 μm; 100 μm). The red arrow indicates calcification in the ultrasound image. Black arrows indicate disorganized collagen fibers. Each panel shows representative images from six rats per group. YN, young normal; YI, young injured; AN, aged normal; AI, aged injured.

Immunohistochemical staining revealed the significantly higher levels of Ki67, CD31 after MCL injury in YI group than AI group (p<0.05; Figure 2A&B). In contrast, the levels of COLIII were increased after MCL injury, with a greater increase in the AI group than YI group (p<0.01). COLI expression tended to decrease after MCL injury, with no significant difference between AI group and YI group (p>0.05). Thus, the ratio of COLI to COLIII remained lower in AI group than that in YI group. TUNEL staining revealed more apoptotic nuclei in the injured MCLs, with the AI group exhibiting more TUNEL-positive cells than the YI group (Figure 2C&D). Our results suggested matrix remodelling was relatively worse in aged rats than young rats.

**Figure 2:**
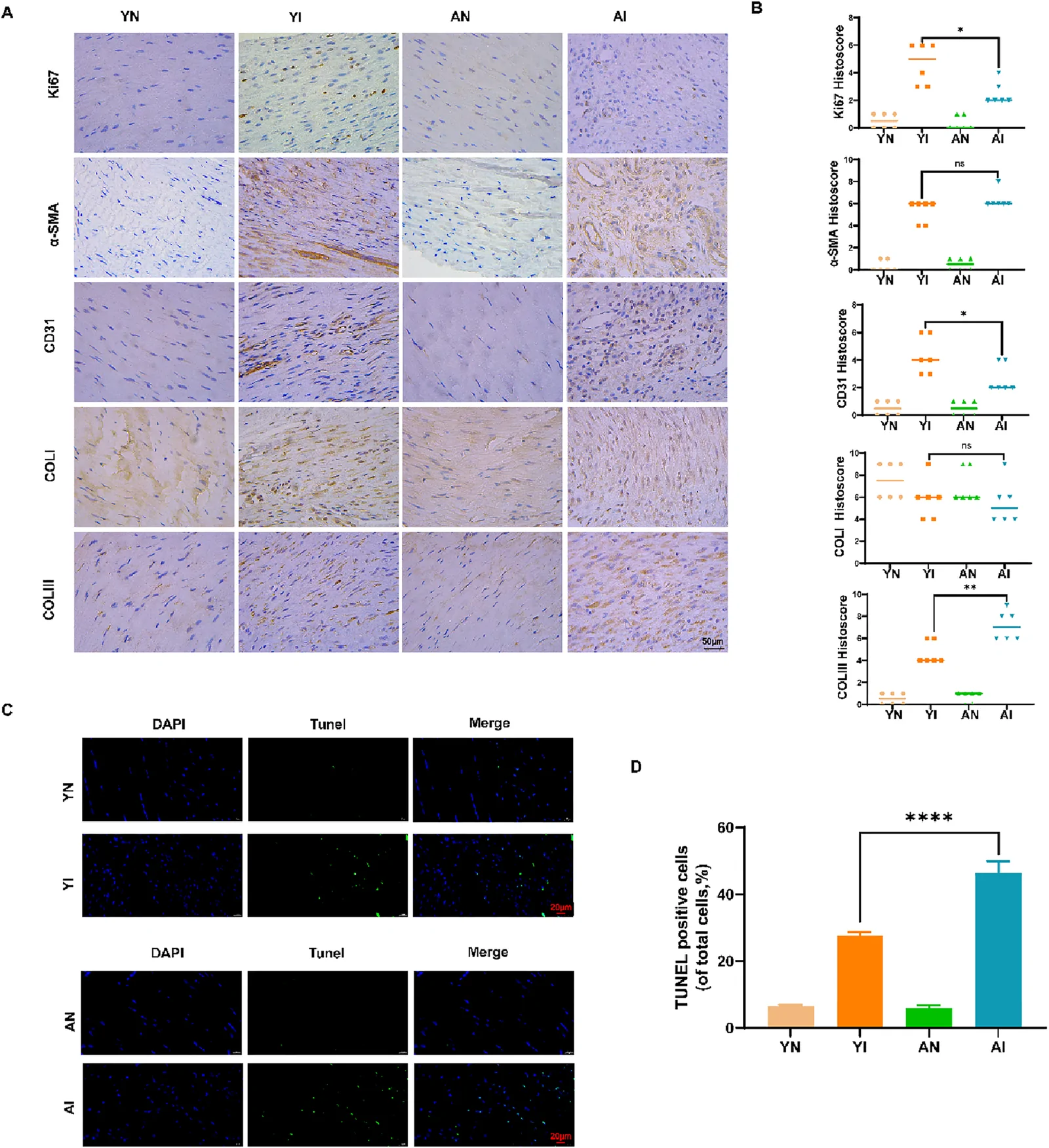
Histological changes of MCL tissues from model rats on postoperative Day 14. (A) Immunohistochemistry staining of MCL tissues (Scale bars: 50 μm). (B) immunoreactivities of protein expression of Ki67, α-SMA, CD31, COLI, and COLIII in MCL tissues assessed using a semiquantitative histological score. At least five random high-power fields from each section were evaluated per panel. (C) apoptosis levels assessed by TUNEL staining (scale bars: 20 μm). (D) quantitative analysis of TUNEL positive cells. At least five non-overlapping microscopic fields from each section were randomly chosen. All quantitative data are presented as mean ± SD. *p<0.05, **p<0.01, and ****p<0.0001; *NS*, no significant difference. YN, young normal; YI, young injured; AN, aged normal; AI, aged injured.

To assess the functional recovery of the MCLs in young and aged rats, biomechanical testing was conducted on postoperative Day 14 (Figure 3). Compared with their respective uninjured controls, both YI and AI groups showed reductions in failure load, stiffness, ultimate stress, and Young’s modulus. However, the decline was much more pronounced in the AI group. These findings indicated that aged rats exhibited worse recovery of ligament function compared with that of young rats.

**Figure 3:**
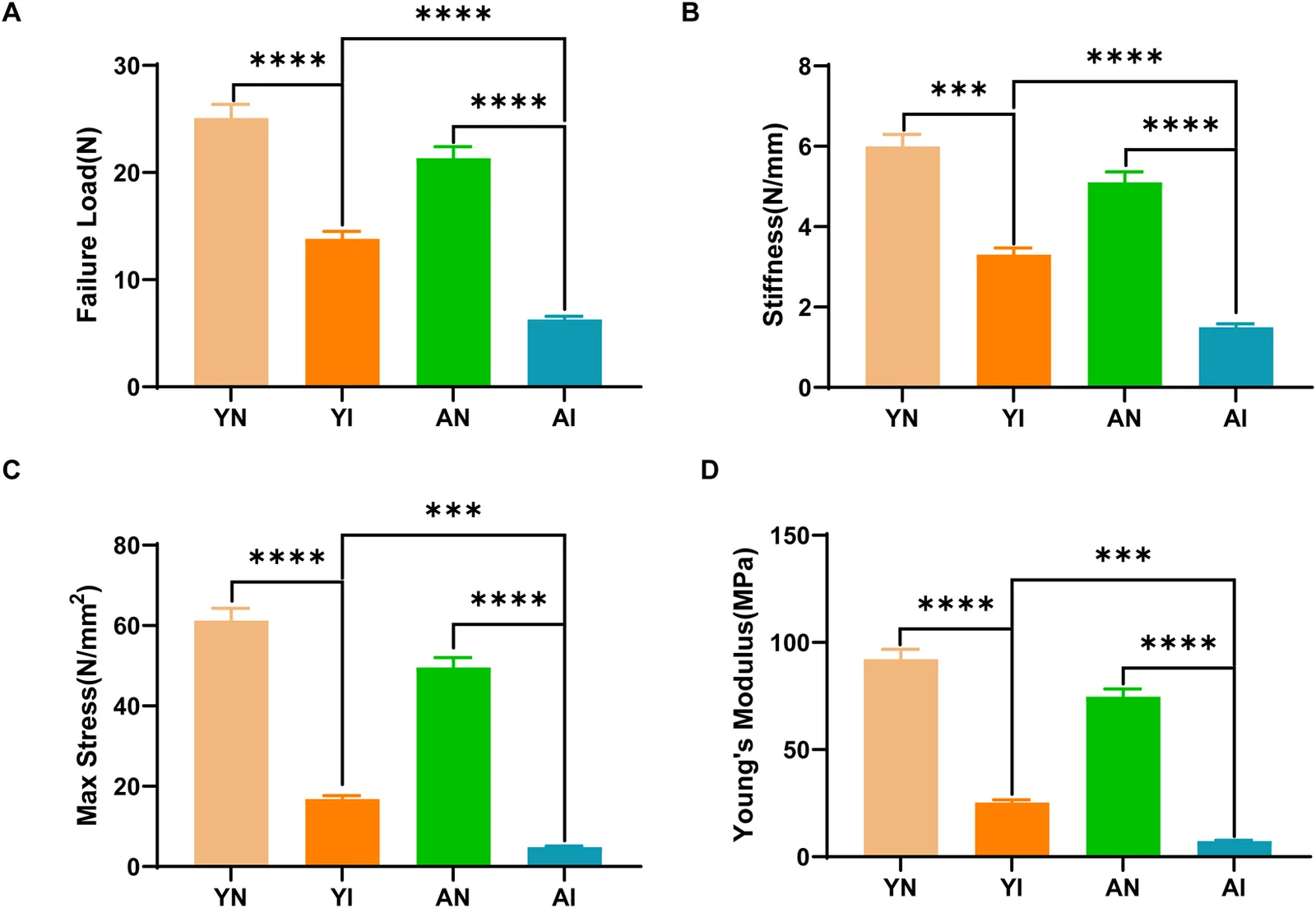
Biomechanical assessment of medial collateral ligaments (MCLs) from model rats on postoperative Day 14. (A) Failure load (B) stiffness (C) maximum stress (D) Young’s modulus. Six rats in each subgroup were used in mechanical testing. All quantitative data are presented as mean ± SD (n=6). ***p<0.001 and ****p<0.0001. YN, young normal; YI, young injured; AN, aged normal; AI, aged injured.

### Impaired MCL repair in aged rats is associated with reduced autophagy in MSCs

To evaluate autophagic activity of MSCs, bone marrow–derived MSCs were isolated from young and aged rats. Flow cytometric profiling revealed that the cultured cells expressed the typical MSC markers, CD44, CD29, and CD90, at >95 % positivity but lacked the CD45 haematopoietic marker, confirming the identity and purity of the isolated MSCs (Figure 4A). Further, the expression of LC3, ATG5, p62, and Lamp1 in MSCs were examined by Western blotting and immunofluorescence staining (Figure 4B–D). The results revealed that lower levels of ATG5 and Lamp1, higher p62 accumulation and the lower LC3-II/LC3-I ratio in MSCs from aged rats than young rats (p<0.05).

**Figure 4:**
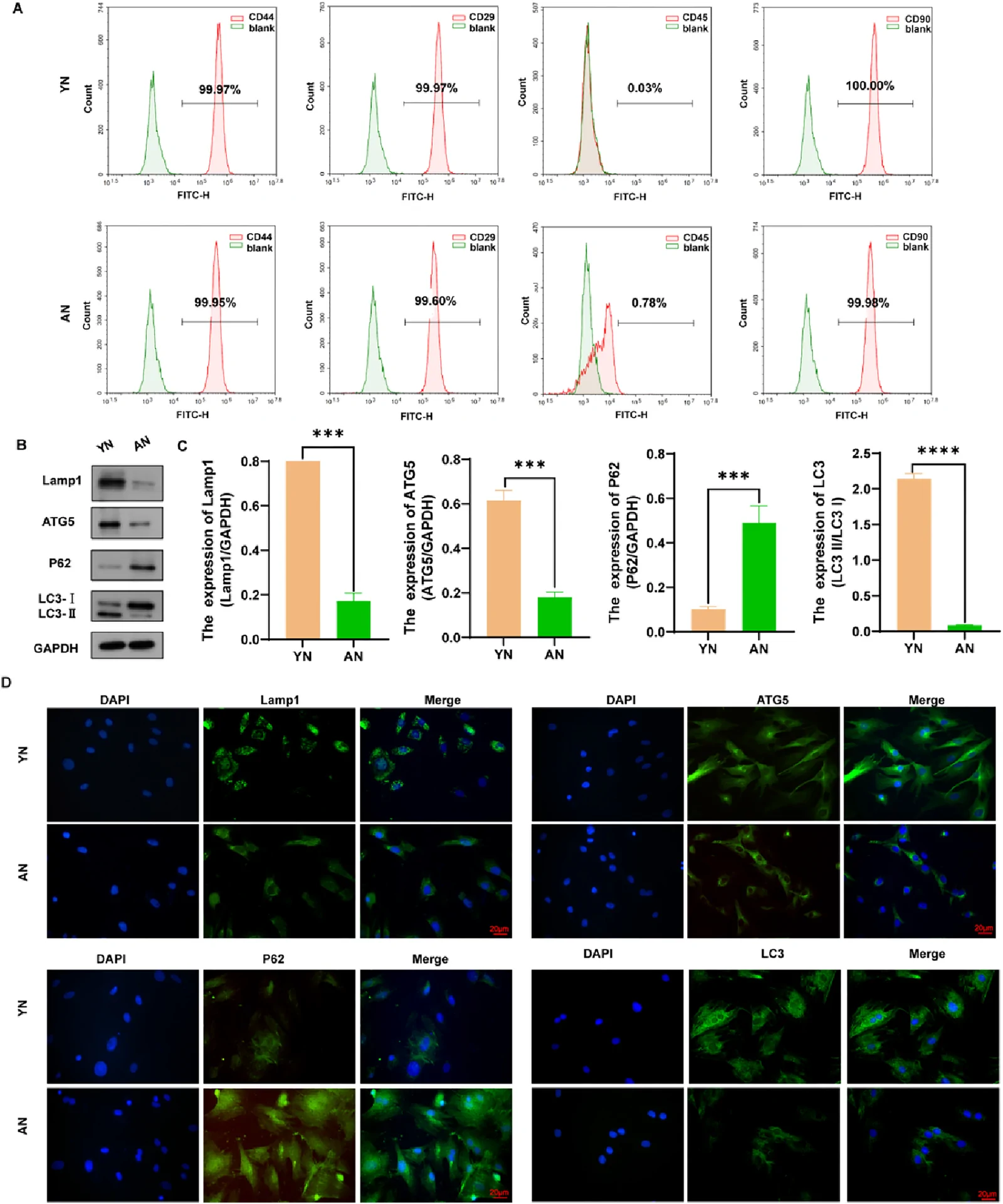
Isolation of bone marrow–derived MSCs from model rats and reduced autophagy in aged model rats. (A) Flow cytometry analysis of surface markers in bone marrow–derived MSCs isolated. (B) Western blotting bands of autophagy-associated proteins (LC3, ATG5, p62, and Lamp1) in MSCs. Experiments were performed and repeated at least three times. (C) the expression levels of autophagy-associated proteins. (D) immunofluorescence staining of LC3, ATG5, p62, and Lamp1 in MSCs from model rats (scale bar: 20 μm). Autophagy-related proteins (green) are shown together with nuclear staining using DAPI (blue). All quantitative data are presented as mean ± SD. ***p<0.001, and ****p<0.0001. YN, young normal; AN, aged normal.

### ATG5-overexpressing MSCs exhibit enhanced autophagic activity and improved MCL repair in aged rats

MSCs were transduced with an ATG5-expressing lentiviral vector to obtain stable cells overexpressing ATG5 (designated MSC^ATG5+^ cells). Western blotting analysis revealed markedly elevated protein levels of ATG5 and LC3 in MSC^ATG5+^ cells compared with those in control MSCs (MSC^vector^ cells) (Figure 5A&B). Immunofluorescence staining consistently revealed stronger fluorescence signals for ATG5 and LC3 in MSC^ATG5+^ cells than in MSC^vector^ cells (Figure 5C). To further evaluate autophagic activity, CQ chase experiments were performed (Figure S1). CQ treatment resulted in accumulation of LC3-II and p62 in both MSCvector and MSC^ATG5+^ cells, supporting effective inhibition of autophagic flux. Moreover, ATG5-overexpressing MSCs exhibited increased LC3-II/LC3-I and reduced p62 levels compared with MSC^vector^ cells, consistent with enhanced autophagic activity.

**Figure 5:**
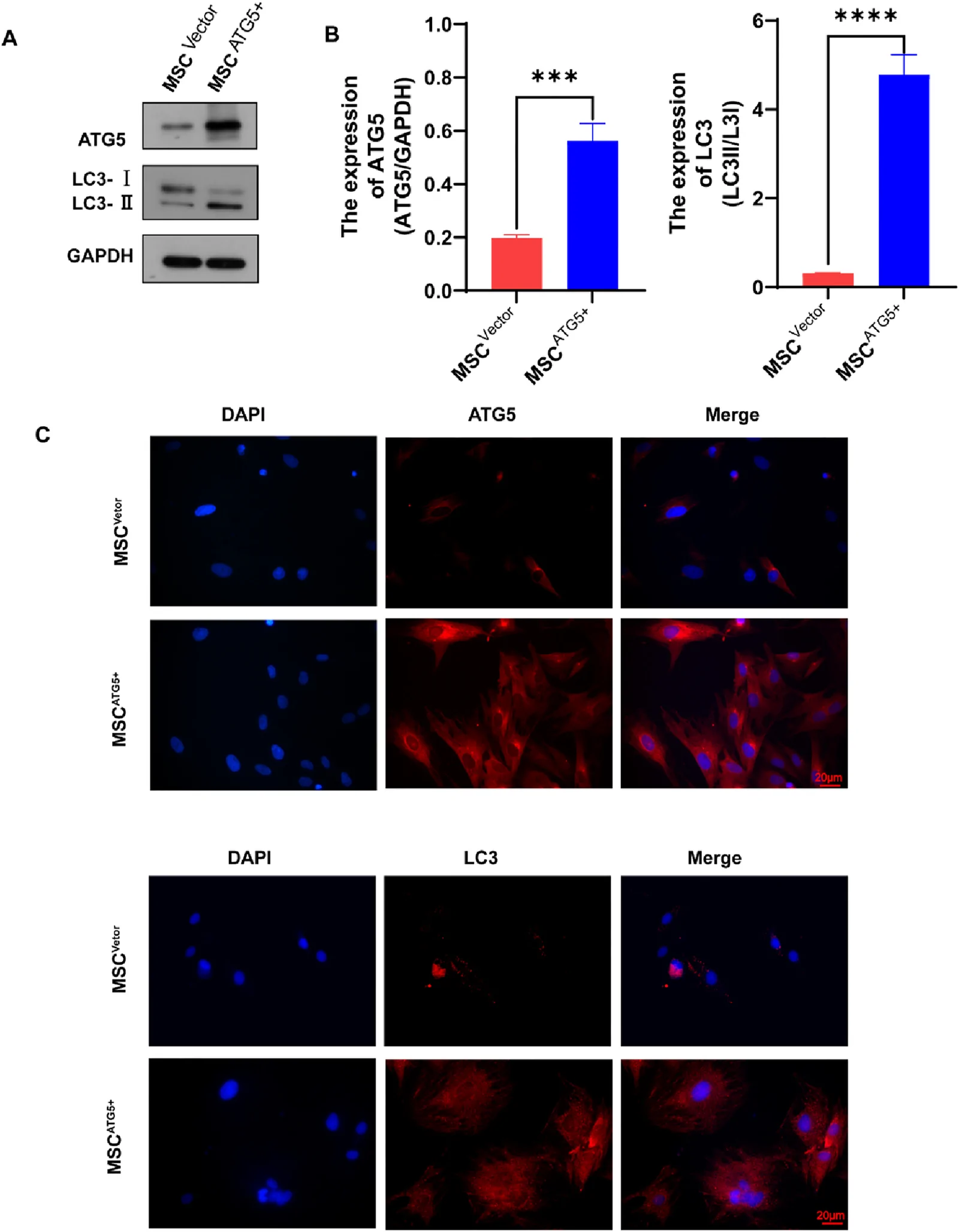
Establishment of stable autophagy-enhanced MSC lines overexpressing ATG5. (A) Western blotting bands of ATG5 and LC3 in MSC^ATG5+^ and MSC^vector^ lines. Experiments were performed and repeated at least three times. (B) the expression levels of ATG5 and LC3 expression in MSC^ATG5+^ and MSC^vector^ lines. (C) immunofluorescence staining of ATG5 and LC3 in MSC^ATG5+^ and MSC^vector^ cells (scale bars: 20 μm). Autophagy-related proteins (red) are shown together with nuclear staining using DAPI (blue). All quantitative data are presented as mean ± SD. ***p<0.001, and ****p<0.0001.

To investigate the reparative potential of ATG5-overexpressing MSCs, MSC^ATG5+^ and MSC^vector^ cells were injected into the aged model rats of MCL (Figure 6). Ultrasound imaging on postoperative Day 14 revealed that the injured MCL regions in the MSC^ATG5+^ group exhibited more uniform echotexture and fewer calcifications compared with those in the MSC^vector^ group (Figure 6A). The results of the histological assessment were in line with these observations. H&E staining revealed that the collagen bundles in the MSC^ATG5+^ group were more regularly arranged, accompanied by a reduction in inflammatory cell infiltration (Figure 6B). Masson’s trichrome staining further confirmed the improved matrix organization, with a greater proportion of mature collagen fibres in the MSC^ATG5+^ group (Figure 6C). Immunohistochemical staining revealed increased expression of Ki67, CD31, and COLI but reduced the expression of COLIII in MCLs from the MSC^ATG5+^ group (Figure 6D&E). Consistently, TUNEL staining revealed a lower frequency of apoptotic nuclei in the MSC^ATG5+^ group than in the MSC^vector^ group (Figure 6F&G). Biomechanical testing revealed better functional recovery of the MSC^ATG5+^ group than the MSC^vector^ group, with significantly higher failure load, stiffness, ultimate stress, and Young’s modulus (Figure 6H).

**Figure 6:**
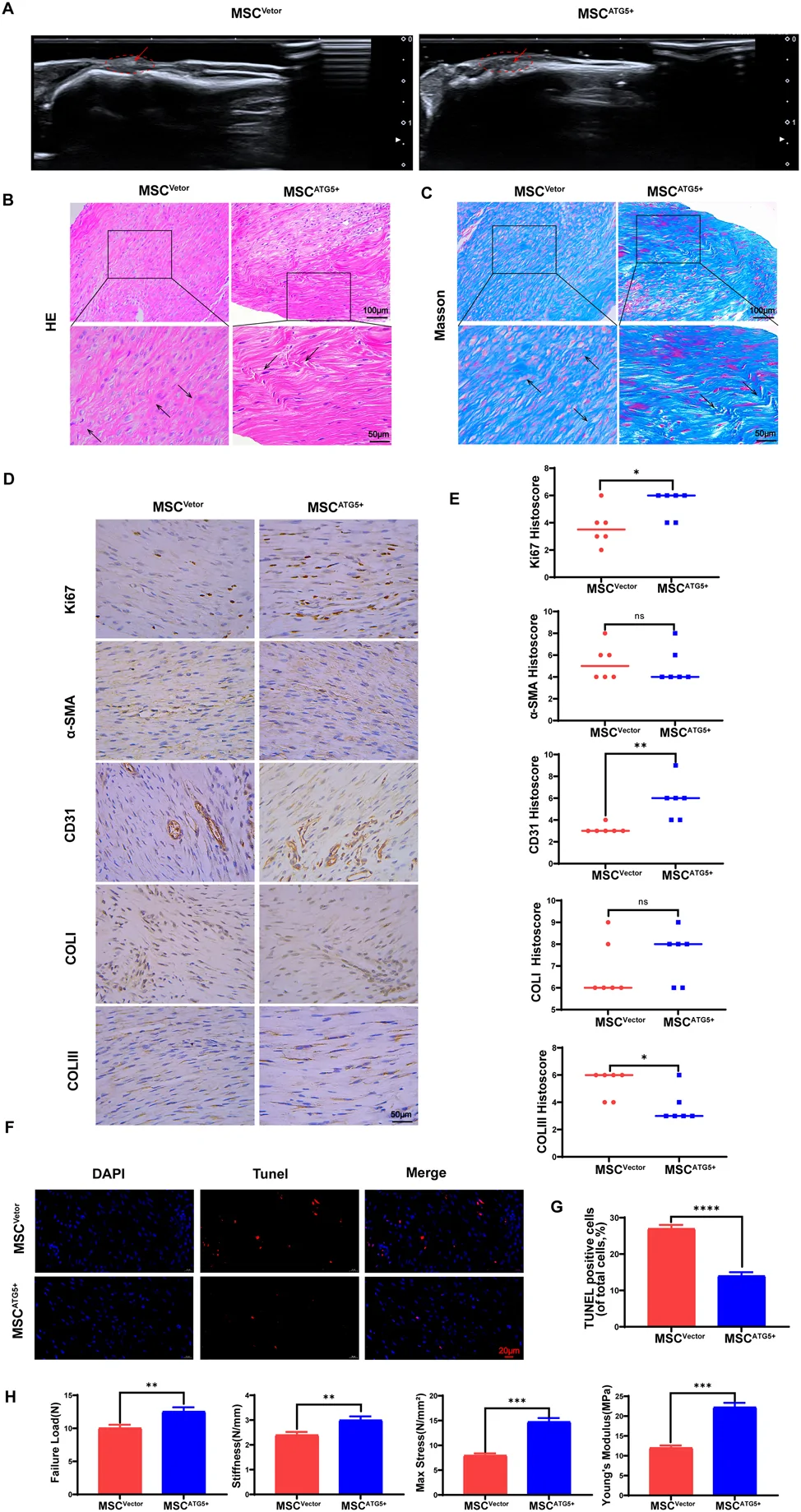
Ligament repair outcomes after transplantation of ATG5-overexpressing MSCs in aged rats on postoperative Day 14. (A) Representative ultrasound images of MCLs. (B) representative H&E staining of MCL tissues (scale bars: 50 and 100 μm). (C) representative Masson’s trichrome staining of MCLs (scale bars: 50 and 100 μm). (D) immunohistochemical staining of the MCL tissues (scale bars: 50 μm). (E) immunoreactivities of protein expression of Ki67, CD31, COLI, COLIII, and α-SMA. (F) Apoptosis levels assessed by TUNEL staining (scale bars: 20 μm). (G) quantitative analysis of TUNEL positive cells. (H) Biomechanical assessment. The red arrow indicates calcification in the ultrasound image. Black arrows indicate disorganized collagen fibers. Six rats in each subgroup were used each experiment. All quantitative data are presented as mean ± SD. *p<0.05, **p<0.01, and ***p<0.001, *NS*, no significant difference.

### ATG5-overexpressing MSC transplantation is associated with an M2-associated macrophage shift during MCL healing

To examine whether ATG5-overexpressing MSC transplantation was associated with changes in the local immune profile, we analysed inflammatory and anti-inflammatory markers in injured tissues. Quantitative RT-PCR demonstrated that the MCLs from the MSC^ATG5+^ group showed lower relative expression levels of CD80 and IL-12, and the higher relative expression levels of CD163 and IL-10 (Figure 7A). Consistently, immunofluorescence staining revealed that CD68+CD206+ M2-like macrophages were more abundant in the MCLs from the MSCATG5+ group, and CD68^+^CD86^+^M1-like macrophages were more abundant in the MCLs from the MSC^vector^ group (Figure 7B&C). ELISA analysis further demonstrated that MCL tissues from the MSC^ATG5+^ group exhibited significantly lower levels of the pro-inflammatory cytokines TNF-α and IL-6, along with higher levels of the anti-inflammatory cytokines IL-4 and TGF-β1, compared with the MSCvector group (Figure 7D).

**Figure 7:**
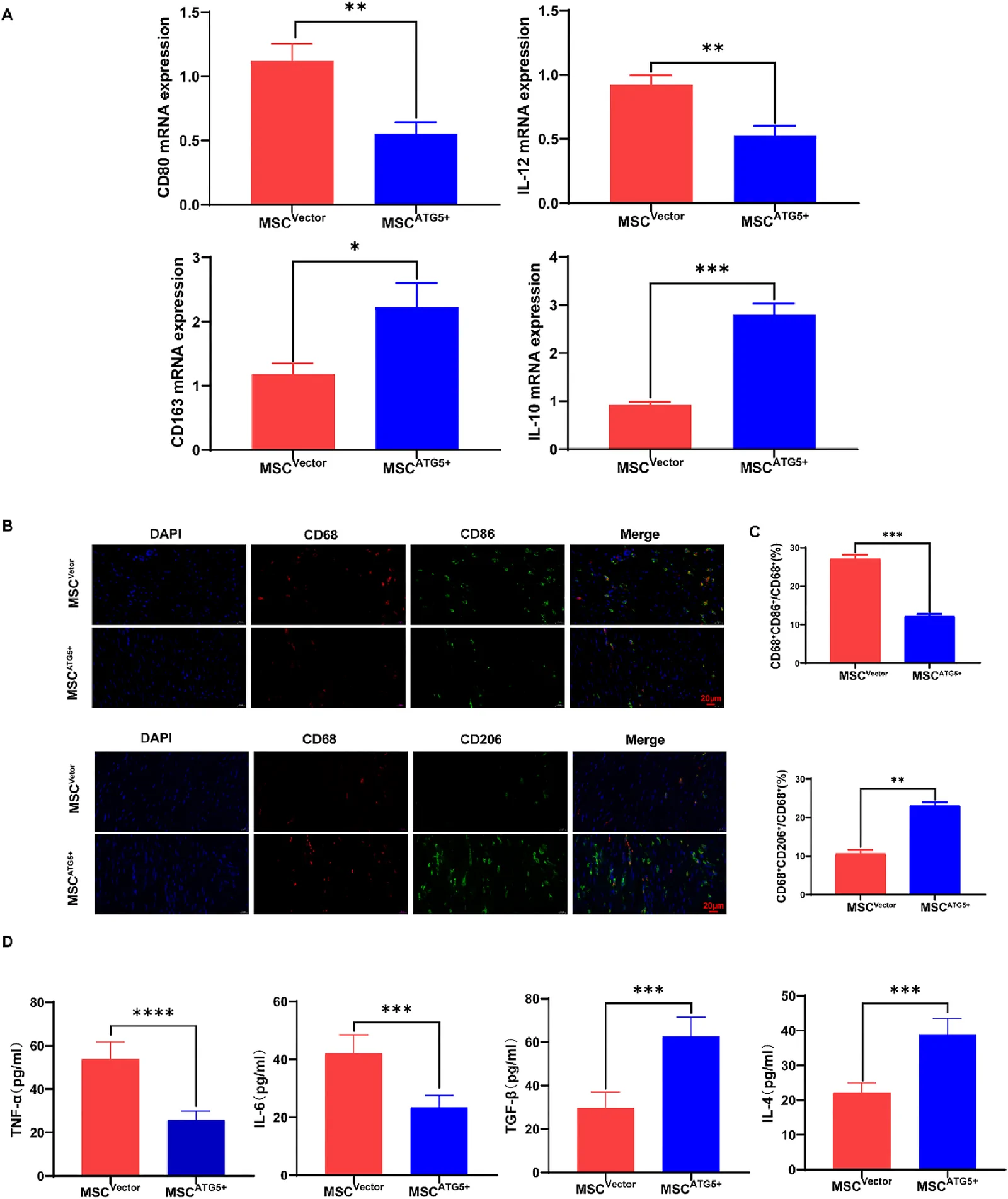
Macrophage-associated changes in medial collateral ligament (MCL) tissue after transplantation of ATG5-overexpressing MSCs. (A) Quantitative RT-PCR analysis of proinflammatory markers (CD80 and IL-12) and anti-inflammatory markers (CD163 and IL-10) in MCLs from aged rats on postoperative Day 14 after transplantation of MSC^vector^ or MSC^ATG5+^ cells. (B) representative immunofluorescence images of CD68 (red), CD86 (green) or CD206 (green), and DAPI (blue) in MCL sections from MSC^vector^ and MSC^ATG5+^ groups (scale bar: 20 μm). (C) quantification of CD68^+^CD86^+^ and CD68^+^CD206^+^ macrophages in MCL tissue from MSC^vector^ and MSC^ATG5+^ groups. (D) ELISA analysis of pro-inflammatory cytokines (TNF-α and IL-6) and anti-inflammatory cytokines (IL-4 and TGF-β1) in MCL tissues from MSC^vector^ and MSC^ATG5+^ groups. Six rats in each subgroup. were used each experiment. all quantitative data are presented as mean ± SD. *p<0.05, **p<0.01, ***p<0.001, and ****p<0.0001.

## Discussion

In the present study, we found that aged rats exhibited significantly impaired ligament healing compared with young rats, which was associated with reduced autophagic activity in aged MSCs. ATG5 overexpression enhanced autophagic activity in aged MSCs, and transplantation of these cells was associated with improved structural and functional repair together with an M2-associated macrophage shift. These findings highlight a potential relationship among aging, MSC autophagic activity, macrophage phenotype, and ligament repair, while the causal links among these processes require further validation.

Autophagy is increasingly recognized as a key regulator in tendon and ligament biology. Previous studies have demonstrated that autophagy regulates tendon homeostasis by controlling type I procollagen secretion and extracellular matrix quality [30]. In addition, autophagy activation has been shown to promote tendon-bone healing by modulating macrophage polarization and suppressing NF-κB–mediated inflammation [31]. Furthermore, autophagy has been implicated in limiting fibroblast activation and reducing tendon adhesion and fibrosis [32]. Together, these findings suggest that autophagy contributes to tendon and ligament repair through coordinated regulation of extracellular matrix remodeling, inflammatory responses, and tissue homeostasis. Mounting evidence confirms that impaired autophagy in MSCs underlies age-related repair deficits across diverse tissues [33]. In bone, diminished MSC autophagy disrupts the osteogenesis-adipogenesis balance, precipitating osteoporosis [20], 34]. In tendon-to-bone healing, reduced autophagy accelerates stem cell senescence, impairing both osteogenic and tenogenic differentiation [35], 36]. Similarly, in the liver, compromised autophagy undermines MSC antifibrotic and immunomodulatory functions, exacerbating fibrosis progression [37]. Collectively, these studies underscore that autophagy dysfunction critically erodes MSC reparative capacity in varied pathological settings. Aligning with these observations, our work extends this paradigm to ligament repair. MSCs from aged rats displayed markedly reduced autophagic activity, evidenced by a lower LC3-II/LC3-I ratio, diminished ATG5 and Lamp1 expression, and p62 accumulation. Functionally, this attenuation correlated with disorganized collagen, impaired matrix remodeling, and suboptimal biomechanical recovery. These findings are consistent with an association between reduced MSC autophagic activity and impaired ligament repair in aging but do not establish autophagy deficiency as the direct cause of the healing deficit. Accordingly, MSC autophagic activity warrants further investigation as a potential therapeutic target for age-related ligament repair.

Based on these mechanistic insights, various strategies have been developed to enhance autophagy and improve tendon and ligament repair in aged individuals [18], 38]. For example, Prim-O-glucosylcimifugin (POG), an active compound derived from *Saposhnikovia divaricata*, protects tendon stem/progenitor cells (TSPCs) from age-related functional decline by suppressing NF-κB activation and inhibiting mTOR signaling, thereby enhancing autophagic activity [25]. Extracellular vesicles derived from young TSPCs (TSPC-EVs) rejuvenate aged tendon cells by restoring autophagy through PI3K/AKT pathway inhibition, with thrombospondin-1 identified as a key effector [35]. Rapamycin, a classical mTOR inhibitor, reverses senescence in TSPCs by enhancing autophagy, partly through promoting HMGB1 translocation from the nucleus to the cytoplasm [39]. In addition, other pharmacological autophagy modulators present attractive options. Spermidine, a natural polyamine autophagy inducer, has demonstrated efficacy in delaying cardiac aging, reducing liver fibrosis, and suppressing age-dependent autophagy decline in human T and B cells [7]. The PPARδ agonist GW501516 activates autophagy by inhibiting the AKT/mTOR signaling pathway, reducing chondrocyte apoptosis and extracellular matrix degradation in osteoarthritis models [10]. Together, these studies establish autophagy activation as a promising therapeutic strategy for improving tendon and ligament healing in aging. However, these approaches share a common limitation: they modulate autophagy indirectly through pharmacological agents, upstream signaling pathways, or paracrine factors, which may lack specificity and confer off-target effects. For instance, systemic rapamycin administration carries risks of immunosuppression, metabolic side effects, and potential impairment of mTOR-dependent anabolic processes essential for tissue repair [40].

In the present study, ATG5 overexpression provided a direct genetic strategy to enhance autophagic activity in aged MSCs and was associated with improved ligament repair after transplantation. As a key regulator of autophagy, ATG5 plays a critical role in autophagosome formation and is essential for maintaining autophagic flux [22] Previous studies have demonstrated that ATG5 overexpression can stably enhance autophagic activity in both cellular and animal models, including the use of ATG5-deficient mice as models of impaired autophagy [41] and lentivirus-mediated ATG5 overexpression to increase autophagic flux [42]. In our study, lentiviral-mediated ATG5 upregulation enhanced autophagic activity in MSCs, while transplantation of these cells was associated with increased Ki67 and COL I expression, more organized collagen deposition, and superior biomechanical properties. Because ATG5 is a core autophagy-related protein, its overexpression provides a targeted means of manipulating autophagic activity; however, ATG5 also has autophagy-independent functions [22], and the present data do not establish that the therapeutic effects are exclusively autophagy-dependent. Consistent with this interpretation, ATG5 overexpression increased autophagic flux, which paralleled improved collagen organization, elevated Ki67 (proliferation marker) and CD31 (angiogenesis marker) expression, reduced apoptosis, and superior biomechanical recovery. Similar benefits of autophagy enhancement have been documented in other degenerative conditions: in aged bone marrow-derived MSCs, activating autophagy restores osteogenic potential and alleviates osteoporosis by counteracting senescence and adipogenic drift [20], 34]. In liver fibrosis, MSC autophagy preserves immunomodulatory/antifibrotic functions, suppressing hepatic stellate cell activation and limiting matrix deposition [37]. Similarly, in osteoporotic tendon-to-bone healing models, strategies to increase MSC autophagy reduce senescence and promote osteogenic/tenogenic differentiation, enhancing structural integration [36]. Together with our ligament data, these cross-tissue observations support autophagy as a candidate regulatory pathway that may contribute to the maintenance of MSC function in age-related degeneration.

Beyond intrinsic cellular roles, MSCs exert therapeutic effects primarily via paracrine and immunomodulatory actions. Emerging evidence emphasizes the immune microenvironment as a important regulator of tendon-bone and ligament healing: chronic inflammation perpetuates M1 macrophage dominance and proinflammatory cytokine release, impairing regeneration, whereas MSCs have been reported to favor M2-associated macrophage phenotypes and anti-inflammatory signaling. Thus, modulating this immune milieu with MSCs or their derivatives has emerged as a promising strategy to improve healing [9], 13], 43]. In our study, transplantation of ATG5-overexpressing MSCs was associated with a distinct local immune profile, characterized by lower M1-associated markers (CD80/IL-12/CD86/TNF-α/IL-6) and higher M2-associated markers (CD163/IL-10/CD206/IL-4/TGF-β1), together with lower proinflammatory and higher anti-inflammatory cytokine levels. This M2-associated profile coincided with improved extracellular matrix remodeling and reduced apoptosis. These parallel changes suggest a potential relationship between ATG5-associated autophagic activity, macrophage phenotype, and improved healing; however, they do not establish that M2 polarization is the causal driver of repair.

The immunoregulatory pattern observed in our study is consistent with literature implicating M2 macrophage polarization in tissue regeneration [9], 13], 43]. Our findings suggest that increased autophagic activity within ATG5-overexpressing MSCs may be associated with altered immunomodulatory capacity, although an upstream causal role was not directly tested. This perspective differs from prior studies focusing on how MSCs or their derivatives activate autophagy in macrophages to foster an anti-inflammatory, proregenerative niche. For example, MSC-derived extracellular vesicles alleviate diabetes-associated atherosclerosis by enhancing macrophage autophagy and shifting polarization from M1 to M2 [44]. Similarly, berberine-treated MSC conditioned medium accelerates tooth socket healing via autophagy-dependent M2 polarization [45]. In diabetic nephropathy, MSCs transfer functional mitochondria to macrophages, where PGC-1α/TFEB-mediated autophagy restores lysosomal activity and promotes M2 polarization, mitigating renal injury [46], 47]. Moreover, TNF-α-licensed MSC exosomes on titanium surfaces enhance osseointegration in type 2 diabetes by inducing macrophage autophagy/M2 polarization via PI3K/AKT/mTOR inhibition [48]. Collectively, these studies highlight macrophage autophagy as an important component of MSC-associated immunomodulation and repair. In contrast, our study focuses on autophagic activity within MSCs themselves. We observed that ATG5 overexpression increased autophagic flux in MSCs and that transplantation of these cells was associated with improved ligament repair and an M2-associated macrophage shift. The directionality of this relationship remains uncertain, because the M2-associated shift could contribute to tissue repair or arise secondarily from an improved local tissue environment. These complementary observations support a potential interaction between MSC autophagic activity and macrophage responses, but the causal necessity and sufficiency of either pathway require direct testing.

Compared with conventional MSC transplantation or growth factor–based therapies, enhancing intrinsic autophagy in MSCs may provide a more targeted strategy to restore the regenerative and immunomodulatory functions of aged MSCs [11], 12]. Although growth factor–based therapies have shown beneficial effects on tendon and ligament healing, their clinical application remains limited because of high cost, short biological half-life, and limited local retention at the injury site [49]. In addition, the therapeutic efficacy of conventional MSC transplantation may be compromised by age-related declines in MSC function [11], 12]. Therefore, targeting autophagy within MSCs may represent a feasible approach to rejuvenate aged MSCs and enhance their regenerative function. In this study, MSCs were delivered via local periligamentous injection to achieve local delivery at the injury site. Compared with systemic administration, which can result in substantial pulmonary entrapment, local injection may improve exposure at the target site while avoiding the additional complexity of scaffold-based strategies [43], 44], 50]. Previous studies in wound healing models have shown that systemic administration of MSCs results in most cells being trapped in the lungs, with only a small fraction reaching the target tissue, whereas local injection significantly improves cell localization and therapeutic efficacy [50]. While scaffold-based approaches can further enhance cell retention and provide a three-dimensional structure for proliferation and migration [43], 44], 50], our study aimed to specifically evaluate the effect of ATG5-overexpressing MSCs in a controlled setting; therefore, local injection was selected as a practical delivery approach for the present study. These considerations provide context for our delivery strategy and its advantages over alternative approaches.

Despite the promising therapeutic effects observed in this study, several challenges remain for the clinical translation of lentiviral-mediated gene modification strategies [51], 52]. Although lentiviral vectors provide efficient and stable gene delivery, their use raises safety concerns, including the risk of insertional mutagenesis, off-target genetic effects, and potential chromosomal instability. In addition, issues such as optimal cell dosage, timing, and delivery strategies require further investigation. Furthermore, the long-term safety of genetically modified MSCs, including potential tumorigenicity and unintended differentiation, remains to be fully evaluated. Alternative approaches, such as non-viral gene delivery systems or transient modulation of autophagy, exosomal cargo (miRNAs, proteins, lipids) may offer safer and more clinically feasible options. Therefore, while our findings highlight the potential therapeutic value of ATG5-overexpressing MSCs, further studies are required to improve safety, optimize delivery strategies, and evaluate long-term outcomes before clinical translation.

Several limitations warrant acknowledgment. **First,** although CQ chase experiments supported enhanced autophagic activity in ATG5-overexpressing MSCs, the potential autophagy-independent functions of ATG5 cannot be fully excluded, and the lack of complementary genetic rescue or pharmacological inhibition strategies limits definitive attribution of the observed therapeutic effects to autophagy. **Second, although an M2-associated macrophage shift was observed together with improved ligament healing, the causal contribution of macrophage polarization was not directly tested through macrophage depletion or signaling-blocking approaches. Accordingly, the observed M2-associated shift may represent a contributor to repair or a downstream consequence of an improved local tissue environment. Third,** flow cytometric analysis of enzymatically digested ligament tissue was not performed; therefore, macrophage subpopulations could not be quantified at the single-cell level using a comprehensive gating strategy. Although the combined immunofluorescence, qRT-PCR, and ELISA findings supported an M2-associated shift, tissue heterogeneity, field-selection bias, and marker co-expression cannot be completely excluded. Moreover, *in vitro* macrophage co-culture, Transwell, and conditioned-medium experiments were not performed; therefore, whether soluble factors or extracellular vesicles released by MSC^ATG5+^ cells are sufficient to induce M2 macrophage polarization remains unclear. The specific paracrine mediators involved, including cytokines, chemokines, and extracellular-vesicle cargo, were not characterized. **Fourth,** treatment-side allocation in Phase II was fixed, with MSC^ATG5+^ cells administered to the right limb and MSC^vector^ cells to the left limb, rather than being randomized or counterbalanced. Therefore, potential side-specific bias cannot be completely excluded, despite the paired statistical analysis, standardized bilateral procedures, and blinded outcome assessment. **Fifth,** only a single early healing timepoint (postoperative Day 14) was evaluated in the present study, limiting assessment of the long-term durability, maturation, and remodeling of the repaired ligament. Consequently, whether the beneficial effects associated with ATG5-overexpressing MSC transplantation persist during later functional recovery remains to be determined. **Sixth,** *in vivo* tracking of transplanted GFP-labeled MSCs was not performed; therefore, the localization, survival, persistence, and migration of the transplanted cells within the injured ligament remain unclear. Consequently, the relative contributions of transient paracrine signaling, sustained local cell persistence, and direct engraftment to the observed therapeutic effects cannot be determined from the present study. **Seventh,** the absence of no-cell and young MSC transplantation control groups limits our ability to distinguish the baseline therapeutic effects of MSC transplantation from the additional benefits associated with ATG5 overexpression and to determine the extent to which ATG5 overexpression restores youthful regenerative function in aged MSCs. Furthermore, although the passage number, lentiviral multiplicity of infection, transplantation dose, and ATG5 protein overexpression were documented, cell viability at the time of transplantation, the longitudinal stability of ATG5 expression, and potential off-target cellular effects, including senescence-related changes, cell-cycle alterations, proliferation changes, and secretome profiles, were not evaluated. Formal release criteria for the transplanted MSC product were also not prespecified, which limits comprehensive assessment of cell-product quality, consistency, and translational reproducibility. **The last**, our surgically transected MCL model represents acute traumatic injury rather than the chronic degenerative changes characteristic of age-related tendinopathy in humans. Only male rats used in study to eliminate hormonal cycle variability, may limit generalizability to female patients, who experience higher rates of certain ligament injuries. Rat ligaments heal with greater intrinsic capacity than human ligaments, potentially overestimating translatable efficacy. As this work used a rodent model, extrapolation to human ligament injuries requires caution; future validation in large animals and clinical cohorts is essential. Future studies incorporating long-term follow-up, dynamic cell tracking, randomized treatment allocation, larger cohorts, chronic degenerative models, and both sexes are warranted to further validate the translational relevance of this strategy.

## Conclusions

This study shows that impaired ligament healing in aged rats is associated with reduced autophagic activity in MSCs. ATG5 overexpression enhanced autophagic activity in aged MSCs, and transplantation of ATG5-overexpressing MSCs was associated with improved structural and functional ligament repair and an M2-associated macrophage shift. These findings support a potential relationship among MSC autophagic activity, macrophage phenotype, and ligament healing, while the causal dependence of the therapeutic effects on autophagy and macrophage polarization remains to be established. ATG5-modified MSCs therefore warrant further investigation as a strategy for age-related ligament repair.

## Supplementary Material

Supplementary Material
