# Supplementary material for: ATG5-overexpressing MSCs improve ligament healing with an associated shift toward M2 macrophage polarization in aged rats
Source: Open Med (Wars). 2026 Oct 12;21(1):20261545. doi: 10.1515/med-2026-1545 (PMC13656048; doi:10.1515/med-2026-1545)
Supplement: Supplementary file 1 [file j_med-2026-1545_suppl_001.docx]

**Supplementary FIGURE S1**

**FIGURE S1. Chloroquine (CQ) treatment inhibits autophagic activity in MSCs and attenuates ATG5-induced autophagy.** (A) Cell viability of MSCs following treatment with different concentrations of CQ (0, 1, 2, 5, 10, 25, 50, and 100 μM) for 4 h, as determined by CCK-8 assay. (B) Representative western blot images of LC3 in MSCs treated with different concentrations of CQ for 4 h. (C) Quantification of the LC3-II/LC3-I ratio in MSCs treated with different concentrations of CQ for 4 h. (D) Representative western blot images of LC3 and p62 in MSC vector and MSCATG5+ cells with or without CQ treatment (25 μM, 4 h). (E) Quantification of the LC3-II/LC3-I ratio and relative p62 expression in MSCvector and MSCATG5+ cells with or without CQ treatment (25 μM, 4 h).

**Supplementary TABLE S1**

**TABLE S1**. Primer sequences used for real-time quantitative PCR

| CD80 | Forward Primer | ACATCGCTAACAACTACTCC |
| --- | --- | --- |
|  | Reverse Primer | CTCTGACAGACAGCTCCAC |
| CD163 | Forward Primer | GATTGCCCTATGACTGCT |
|  | Reverse Primer | GGACCGAAGATGATGAACTA |
| IL 12 | Forward Primer | GCTGGTGTCTCCACTCAT |
|  | Reverse Primer | CTTTCCAGAGCCTATGACT |
| IL-10 | Forward Primer | CAGTCAGCCAGACCCACAT |
|  | Reverse Primer | GGCAACCCAAGTAACCCT |
| GAPDH | Forward Primer | ATGACATCAAGAAGGTGGTGAAGCAGG |
|  | Reverse Primer | GCGTCAAAGGTGGAGGAGTGGGT |
